# Factors Influencing the Postoperative Flexion Angle in Cruciate-Sacrificing Rotating Platform of Total Knee Arthroplasty

**DOI:** 10.7759/cureus.66915

**Published:** 2024-08-15

**Authors:** Daisuke Matsuoka, Yusuke Inagaki, Yuya Mawarikado, Munehiro Ogawa, Munehito Seko, Tadashi Fujii, Yasuhito Tanaka

**Affiliations:** 1 Department of Orthopaedic Surgery, Kashiba Asahigaoka Hospital, Nara, JPN; 2 Department of Rehabilitation Medicine, Nara Medical University, Nara, JPN; 3 Department of Medicinal Biology of Thrombosis and Hemostasis, Nara Medical University, Nara, JPN; 4 Department of Sports Medicine, Nara Medical University, Nara, JPN; 5 Department of Orthopaedic Surgery, Nara Medical University, Nara, JPN

**Keywords:** adl (activities of daily living), knee alignment, proms, knee range of motion (rom), tka

## Abstract

Background: Various factors affect the improvement of range of motion (ROM) after total knee arthroplasty (TKA). However, there are few reports specific to cruciate-sacrificing rotating platform (CSRP) TKA. In this study, factors affecting postoperative ROM improvement of CSRP TKA were investigated.

Methods: The study included 79 patients with knee osteoarthritis who underwent unilateral CSRP TKA at our institution. The group with an improvement of 5° or more (Δflexion angle) than the preoperative was defined as the good Δflexion group (38 knees), and that with less than 5° was defined as the poor Δflexion group (41 knees). The assessments were performed one day before and one year after surgery. Factors including rest and walking pain, knee flexion and extension angle, isometric knee extension strength, the five subscales of Knee injury and Osteoarthritis Outcome Score (KOOS), α, β, γ and δ angles, femoro-tibial angle (FTA), and condylar twist angle were assessed. Unpaired t-test, Mann-Whitney U test, and Chi-square test were used to test differences between the good and poor Δflexion groups. Multiple logistic regression examined the association between each factor and the dependent variables (good Δflexion or poor Δflexion).

Results: Significant differences in the preoperative knee flexion, postoperative knee flexion, preoperative knee extension, and postoperative knee extension angles, postoperative KOOS pain and activity of daily living, β, ɤ angles were observed between the good and poor Δflexion groups. The model Chi-squared test revealed that the ɤ angle was significantly affected by the Δflexion angle.

Conclusions: With the CSRP TKA, flexion insertion of the femoral component was associated with postoperative flexion ROM improvement.

## Introduction

Various factors influence patient satisfaction after total knee arthroplasty (TKA) [1]. Among these factors, a good postoperative flexion angle is an important factor that contributes to postoperative satisfaction [1]. Conversely, it has been reported that the factors affecting patient satisfaction after TKA surgery are not ROM but the pain and function of Western Ontario and McMaster Universities Osteoarthritis Index (WOMAC) [2]. Postoperative satisfaction varies according to the postoperative flexion ROM obtained, which depends on the optimal alignment (The α, β, γ, and δ angles, and femoro-tibial angle, FTA), ligament balance, and component design.

It has been reported that there is a correlation between the preoperative flexion angle in posterior stabilized (PS) and postoperative flexion angle in males [3] and that lateral laxity is an important factor in cruciate retention [4]. In general, the cruciate-sacrificing rotating platform (CSRP) is considered to be disadvantageous in terms of postoperative flexion range of motion compared to PS [5]. Previous reports have shown that posterior condylar offset (PCO) is important for obtaining a larger postoperative flexion angle in CSRP and for the stability of the femorotibial joint [6].

With regard to Attune CSRP^TM^ (DePuy Synthes, Warsaw, IN), which has a GRADIUS curve design on the femur side to ensure stability in the mid-flexion position, there have been no previous reports investigating the factors on the ROM improvement.

The aim of this study was to determine which factors affect the flexion angle improvement one year after Attune CSRP TKA. Moreover, it was hypothesized that patients with good flexion improvement after TKA would show good short-term patient-reported outcomes (PROMs). This article was previously posted to the Research Square preprint server in July 2023.

## Materials and methods

Ethics

All participants were fully informed of the study, both verbally and in writing, in accordance with the Declaration of Helsinki, and informed consent was obtained from all participants. This study was approved by the Research Ethics Committee of the Kashiba Asahigaoka Hospital (approval number 03-1-016).

Study design and participants

This retrospective longitudinal study aimed to clarify the factors associated with knee flexion angle improvement at 1 year after the Attune CSRP TKA. We retrospectively reviewed 79 patients with osteoarthritis of the knee who underwent unilateral Attune CSRP TKA at our institution between October 2019 and January 2022. The inclusion criteria were as follows: (1) patients who underwent unilateral TKA due to a diagnosis of knee osteoarthritis and were available for follow-up 1 year after surgery; (2) participants using CSRP models; (3) participants who had the ability to ambulate independently or with a T-cane preoperatively and one-year post-operatively; (4) patients scheduled for primary TKA; (5) age 50-90 years; and (6) participants who provided informed consent to participate in the study. The exclusion criteria were as follows: (1) participants diagnosed with rheumatoid arthritis and idiopathic osteonecrosis; (2) participants with neurological or other musculoskeletal diseases that significantly impaired basic movements, such as walking; and (3) participants with severe depression or dementia that would make the evaluation difficult. The 79 included participants were divided into two groups according to the degree to which the knee flexion angle improved postoperatively compared to preoperatively. The group with an improvement in angle of 5° or more from the preoperative value was defined as the good Δflexion group (n=38) and the group with an improvement angle of less than 5° was defined as the poor Δflexion group (n=41).

CSRP and surgical procedures

**Figure 1:**
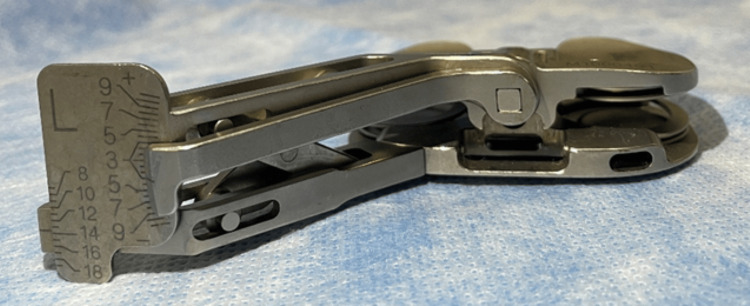
Figure illustrating RTKB Real-Time Knee Balancer® (RTKB) can be used to measure the medial-lateral balance and distance of the extension and flexion gaps.

All patients underwent surgery under general anesthesia following ultrasound-guided sciatic and femoral nerve blocks (20 mL of 0.25% ropivacaine) performed by a trained anesthesiologist [7]. Perioperative antibiotics were administered to patients without renal dysfunction: an intravenous dose of cephazolin 1 g was administered before surgery, followed by an intravenous dose of cephazolin 1 g every eight hours until the day after surgery. Patients with renal impairment with creatinine clearance less than 30 ml/min were given half the dose of cefazolin for the same period as patients without it. To prevent deep vein thrombosis, all patients were wrapped with an elastic bandage from the thigh to the dorsum of the foot after surgery and were encouraged to do plantar dorsiflexion exercises in bed as much as possible. All patients received edoxaban tosilate hydrate 30 mg for one week starting three days after surgery. Preoperative planning was performed using a 3D preoperative-planning software (ZedKnee; Lexi, Tokyo, Japan) in all cases [8]. The coronary alignment of the preoperative plan was mechanical alignment. The osteotomy of the distal femur was flexed along the anterior bowing to avoid anterior notching for sagittal alignment. All patients were placed in the supine position with a tourniquet pressurized at 280 mmHg regardless of blood pressure and deployed using a midline incision and trivector approach. The surgical technique included extension gap first, gap-balancing approach using a spring tensor (Real-Time Knee Balancer®; RTKB;) (Figure 1).

**Figure 2:**
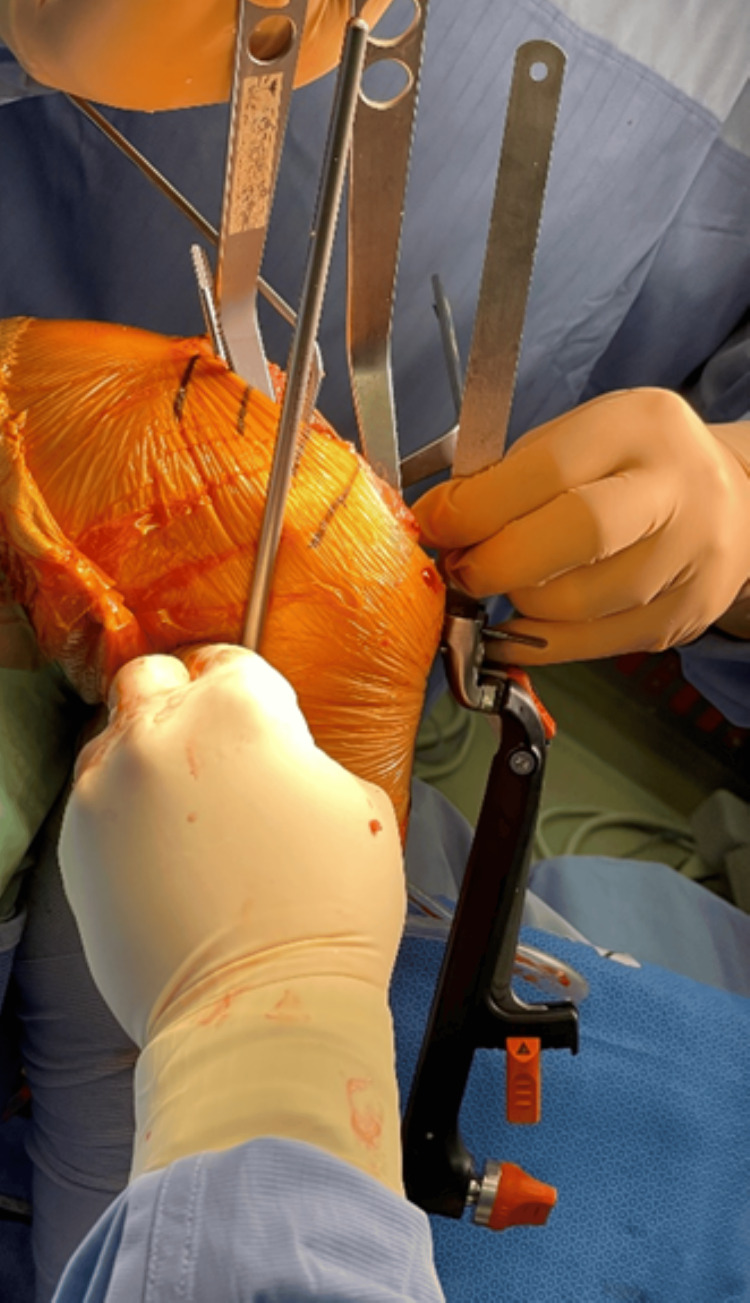
Confirmation of the tibial posterior slope angle using an extramedullary rod and an alignment rod The figure illustrates the confirmation of the tibial posterior slope angle using an extramedullary rod and an alignment rod. An extramedullary rod was first applied parallel to the fibular axis and then a posterior slope angle of 5° was set.

A bony cut of the distal femur was performed using an intramedullary rod. The bony cut of the proximal tibia was performed with an extramedullary tibial guide. Computer-assisted surgery, such as a navigation system, was not used in all cases; only existing devices were used. Therefore, for the intraoperative index when the HKA angle was 0°, we determined the valgus angle and the assumed osteotomy amount obtained in the preoperative 3D planning for the femur and determined that there was no significant difference in the actual osteotomy amount. For the sagittal alignment of the tibia, we set the posterior slope angle in all cases to 5° from the line connecting the lateral malleolus and the fibular head (Figure 2).

**Figure 3:**
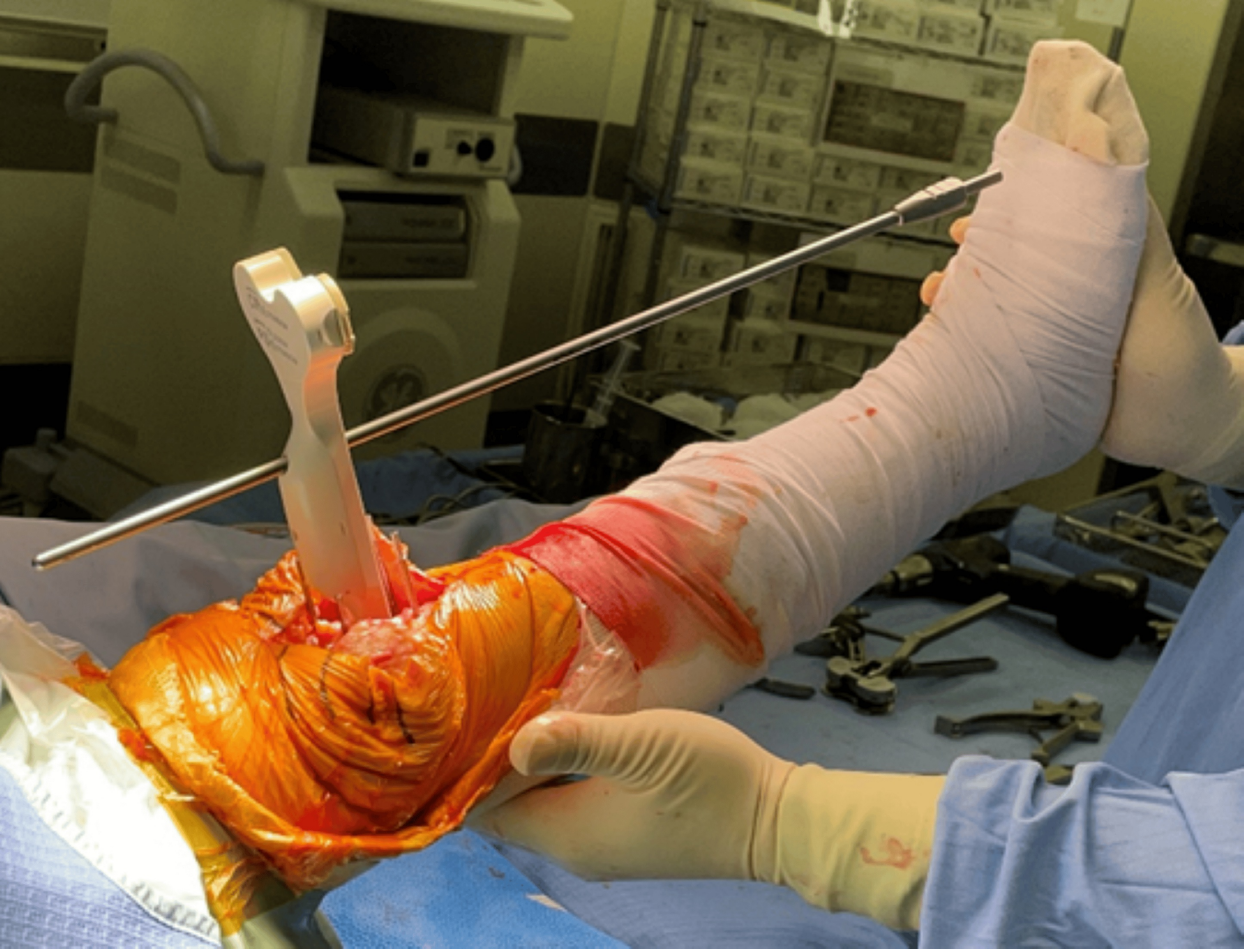
Checking extension alignment using spacer block and alignment rod The figure illustrates checking the extension alignment using a spacer block and an alignment rod. The tibial axis can be confirmed to pass through the middle one-third of the tibial crest.

**Figure 4:**
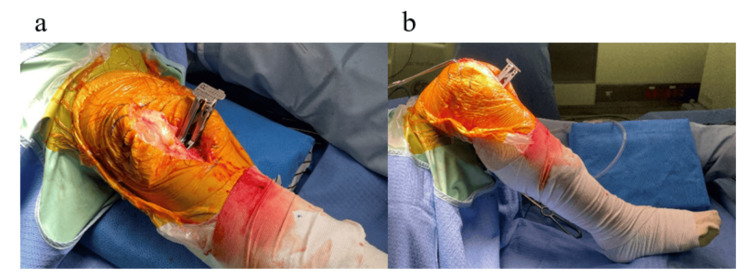
Measurement of extension and flexion gaps using RTKB Measurement of (a) extension gap and (b) flexion gap using Real-Time Knee Balancer® (RTKB). The femur twist angle was also confirmed at 90° of flexion, which is referred to in the subsequent four-sided osteotomy of the femur.

After creating the extension gap, we inserted a spacer block and confirmed that the HKA angle was 0° using an alignment rod (Figure 3). Regarding the point through which the alignment rod passes, the reference of the center of the femoral head was two fingers from the iliac crest [9]. If there was no foot deformation, the center of the ankle joint was set at the second toe and one-third of the center of the tibial axis [10]. After visually confirming the alignment during extension with a spacer block, extension, and flexion gaps were also confirmed with RTKB. Each extension gap at the natural extension position and femoral rotation angle at 90° of flexion were determined using the RTKB (Figure 4). A measured sizing and rotation guide was used to perform a four-sided femoral cut at the femoral rotation angle displayed in the RTKB, and the osteotomy was performed by shifting the ready-made cut block anterior and posterior to the femur so that the gap between extension and flexion was as equal as possible. Then, clearance of the posterior condylar osteophytes, which can impinge on the posterior lip, was performed [11]. The femoral and tibial components were then implanted, with no patella replaced. A mobile insert was used in all the cases.

Outcome assessment

The assessments were conducted in a rehabilitation room in the hospital or in patient rooms. Physical function was assessed by a physiotherapist and baseline information and questionnaires were collected and administered, respectively by a nurse. Patients were assessed a day prior to their scheduled TKA and one year after the surgery. The outcome of interest was the degree of improvement in the knee flexion angle (Δflexion angle) from preoperative to one-year postoperatively.

Preoperative factors

**Figure 5:**
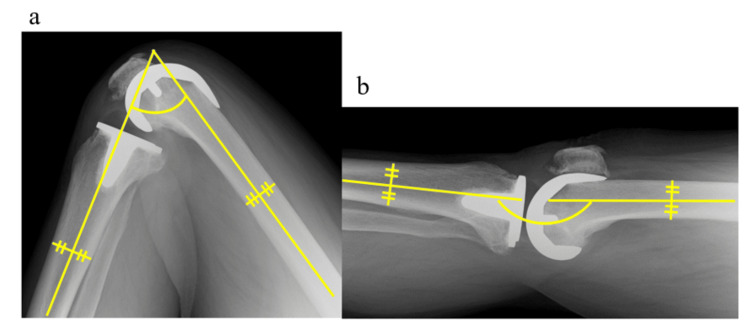
A representative radiographic image (sagittal view) of the knee joint All patients were imaged in full extension and full flexion of the knee joint in the supine position on the sagittal radiographic view. The angle between the line passing through the center of the tibia and that of the femur was measured. (a) shows the maximum flexion angle and (b) shows the maximum extension angle. For this paper, (b) is represented by an angle of 180 minus (b).

Factors including age, height, weight, body mass index, sex, rest and walking pain, knee flexion and extension angle on the affected side, isometric knee extension strength (IKES) on the affected side, five subscales of the Knee injury and Osteoarthritis Outcome Score (KOOS), and condylar twist angle obtained from preoperative CT transverse view were collected. Rest and walking pain were assessed using a visual analog scale (VAS). VAS is a measurement tool used to quantify the subjective feeling of pain in patients. The VAS score ranges from 0 to 10 points, with 0 representing no pain and 10 representing excruciating pain. Passive knee angles were measured during maximum flexion and extension using radiography (Figure 5). IKES was measured using a handheld dynamometer (μ-tas F1, ANIMA, Tokyo, Japan) with the participant in a seated position and the knee at 90° flexion [12]. The reliability and validity of this measurement method have been previously demonstrated [13]. The dynamometer was placed perpendicular to the leg, just above the malleolus. Each participant was instructed to push against the dynamometer while attempting to straighten the knee, gradually increasing the force to maximize the voluntary effort. The maximum effort was then maintained for an additional 3 s.

During all tests, the dynamometer was stabilized by a belt and the examiner's hands. The maximum IKES was measured twice, and the mean value (kg) was calculated. The KOOS measures functional outcomes using the following five subscales: symptoms (seven items), pain (nine items), activities of daily living (ADL) function (17 items), sports and recreational function (five items), and quality of life (QOL) (four items). Each of the five subscale scores is calculated as the sum of the included factors. The scores are then converted to a 0-100 scale, with 0 representing extreme knee problems and 100 representing no knee problems, as is common in orthopedic scales [14]. A Likert scale was used. All factors had five possible answer options ranging from 0 (no problems) to 4 (extreme problems), with scores between 0 and 100 representing the percentage of the total possible score. In addition to the separate analysis and interpretation of the five subscales, an aggregate score was calculated. The reliability and validity of this assessment have been previously reported [15].

Radiological assessment

**Figure 6:**
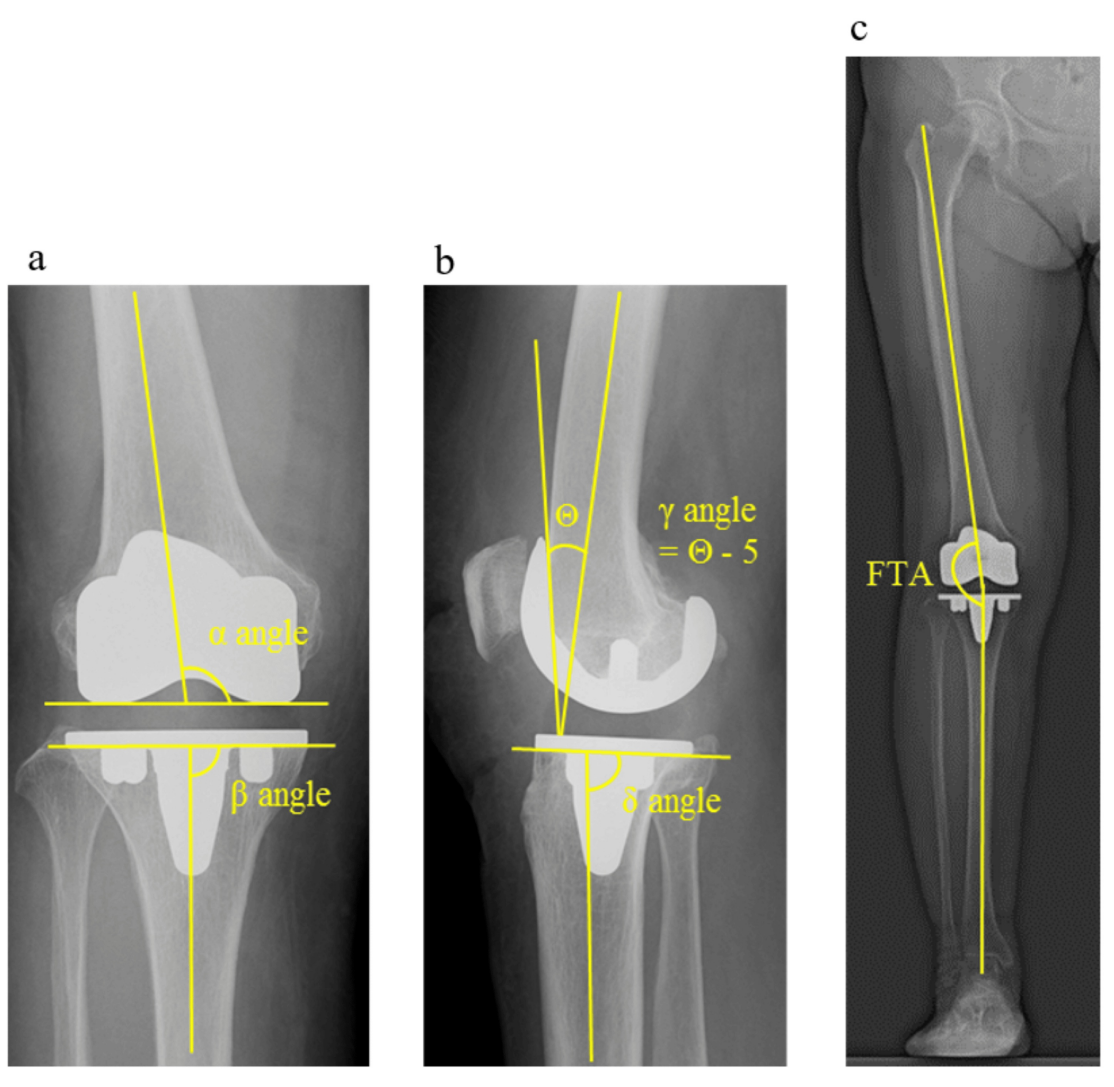
Postoperative radiological evaluation images Postoperative radiological evaluation included (a) α (femoral valgus) angle, β (tibial varus) angle, (b) γ (femoral flexion) angle, δ (tibial posterior slope) angle, and (c) femoro-tibial angle (FTA). Θangle is the angle consisting of the femoral axis and the anterior flange of the femoral component.

The α, β, γ and δ angles and femoro-tibial angle (FTA) were assessed by early postoperative X-ray images (Figure 6). The α angle was measured between the line across the lower edge of the femoral component and the axis of the femoral shaft. The femoral component was typically implanted 5-7° valgus to the anatomical axis of the femur, which is the amount necessary to restore the neutral mechanical axis of the limb [16]. The β angle was measured between the line across the base of the tibial plate and the axis of the tibial shaft. The tibial component was neutrally positioned at 90° [17]. The γ angle was measured between the anterior femoral cortex and the inner anterior part of the femoral component. Because the anterior flange was opened at 5°, the femoral component axis was measured considering the angle in the Attune CSRP. The ideal γ angle recommended in some studies varies between 0-10° [18]. The σ angle was measured between the line across the base of the tibial plate and the axis of the tibial shaft. The ideal σ angle was 86° [19]. The FTA is the lateral angle between the distal femoral and proximal tibial axes (i.e., <180° and >180°, corresponding to valgus and varus alignments, respectively).

Outcomes at one year postoperatively

Factors including rest and walking pain, knee flexion and extension angles on the affected side, IKES on the affected side, and the five KOOS subscales were assessed.

Postoperative protocol

The length of hospital stay was two weeks according to the hospital protocol, with six days of physiotherapy, 40-60 minutes a day. Physiotherapy was started on the first postoperative day, and if possible, gait exercises with a walker and knee flexion exercises were initiated. Once discharged from the hospital, weekly physiotherapy sessions (40 minutes per session) were provided for approximately two to three months. Once physiotherapy was completed at two to three months post-operatively, as agreed upon by the participant and the attending doctor, the patient resumed daily routine activities at home until the one-year postoperative assessment. In this study, only patients who underwent rehabilitation in the same program were selected [20].

Statistics

Descriptive data are presented as the number of cases and mean with standard deviation (SD). For all analyses, the significance level was set at 5%. All statistical analyses were performed using SPSS Statistics for Windows, Version 26.0 (IBM Corp., Armonk, NY). The Shapiro-Wilk test was used to determine the normality of the data distribution.

To test for differences between the good and poor Δflexion groups, the unpaired t-test, Mann-Whitney U test, and Chi-square test were used. Multiple logistic regression was performed to examine the association between each factor and the dependent variable (i.e., good Δflexion or poor Δflexion). The independent variables were the β angle and the ɤ angle. The Hosmer-Lemeshow test was performed to determine whether the analysis results significantly fit the actual data. To test for differences between the groups preoperatively and at one year postoperatively, the paired t-test and Wilcoxon signed-rank test were used.

## Results

Table 1 summarizes the participants’ descriptive characteristics and the results of the unpaired t-test, Mann-Whitney U test, and Chi-square test. Significant differences in the preoperative knee flexion angle (p=0.001), postoperative knee flexion angle (p=0.021), preoperative knee extension angle (p=0.009), postoperative knee extension angle (p=0.010), postoperative KOOS pain (p=0.028), and postoperative KOOS ADL (p=0.007), β angle (p=0.033), ɤ angle (p=0.006), were observed between the good and poor Δflexion groups.

**Table 1:** Participant’s demographic characteristics and statistical data ADL: activities of daily living; BMI: body mass index; FTA: femorotibial angle; IKES: isometric knee extension strength; KOOS: knee injury and osteoarthritis outcome score; QOL: quality of life ^a^unpaired t-test, ^b^Mann–Whitney U test, ^c^Pearson’s chi-square test.

Characteristics	Good Δflexion group (n = 38)	Poor Δflexion group (n = 41)	p-value
Age, years	72.37 ± 8.68	73.73 ± 8.70	0.325^b^
Height, cm	156.63 ± 9.29	156.44 ± 9.36	0.928ª
Weight, kg	63.49 ± 11.46	65.49 ± 13.18	0.864^b^
BMI, kg/m²	25.71 ± 2.77	26.58 ± 3.52	0.229ª
Gender			0.057^c^
Female	21	31	
Male	17	10	
Operative side			0.552^c^
Right	22	20	
Left	16	21	
α angle, °	96.74 ± 2.43	96.39 ± 2.06	0.535^b^
β angle, °	91.11 ± 2.04	90.24 ± 1.55	0.033^b^
γ angle, °	4.47 ± 3.27	2.57 ± 2.67	0.006^a^
δ angle, °	84.79 ± 2.86	85.20 ± 2.37	0.493ª
External rotation angle, °	4.45 ± 1.98	4.54 ± 2.26	0.755^b^
FTA, °			
Pre-operation	180.42 ± 8.17	182.59 ± 6.12	0.130^b^
One year postoperatively	172.08 ± 3.14	173.37 ± 2.86	0.060^a^
Rest pain, mm			
Pre-operation	20.63 ± 25.89	16.17 ± 15.41	0.820^b^
One year postoperatively	10.97 ± 17.88	9.32 ± 19.96	0.108^b^
Walking pain, mm			
Pre-operation	51.97 ± 30.84	48.63 ± 24.78	0.596^a^
One year postoperatively	17.74 ± 21.02	13.12 ± 21.16	0.080^b^
Knee flexion angle, °			
Pre-operation	127.24 ± 16.09	141.32 ± 9.40	0.001^a^
One year postoperatively	130.11 ± 11.97	123.85 ± 11.58	0.021^a^
Knee extension angle, °			
Pre-operation	−3.18 ± 5.49	−0.64 ± 8.74	0.009^b^
One year postoperatively	1.54 ± 6.07	5.05 ± 7.40	0.010^b^
IKES, kgf			
Pre-operation	16.16 ± 10.56	16.10 ± 8.77	0.620^b^
One year postoperatively	18.63 ± 8.78	20.12 ± 10.25	0.413^b^
KOOS Symptoms, %			
Pre-operation	64.38 ± 19.80	62.20 ± 17.09	0.600^a^
One year postoperatively	77.26 ± 12.60	81.18 ± 16.83	0.095^b^
KOOS Pain, %			
Pre-operation	54.90 ± 22.81	53.93 ± 16.59	0.831^a^
One year postoperatively	78.00 ± 15.80	85.16 ± 14.28	0.028^b^
KOOS ADL, %			
Pre-operation	62.89 ± 19.68	63.49 ± 15.77	0.600^a^
One year postoperatively	76.01 ± 14.17	84.54 ± 12.78	0.007^b^
KOOS Sport/Rec, %			
Pre-operation	28.16 ± 24.89	27.68 ± 24.06	0.832^b^
One year postoperatively	43.82 ± 25.51	49.27 ± 29.30	0.340^b^
KOOS QOL, %			
Pre-operation	34.54 ± 22.07	33.23 ± 19.64	0.878^b^
One year postoperatively	53.62 ± 18.36	60.06 ± 24.76	0.191^a^

The results of the multiple logistic regression analyses are shown in Table 2. The model Chi-squared test (significant at p < 0.01) revealed that the ɤ angle (B = -0.187, p=0.037, odds ratio (OR) = 0.830) was significantly associated with Δflexion angle. The result of the Hosmer-Lemeshow test was not significant at p=0.984, and the fit of the regression equation was good.

**Table 2:** Multiple logistic regression analysis for factors affecting Δflexion angle Regression equation: ln (p/1−p) = −0.183 (β angle) −0.187 (γ angle) + 17.353. Model x2, p < 0.01. Hosmer-Lemeshow test, p = 0.982.

Angle	β	Standard error	Wald	p-value	Odds ratio (95% CI)
β	−0.183	0.150	1.490	0.222	0.832 (0.620–1.117)
γ	−0.187	0.090	4.352	0.037	0.830 (0.696–0.989)
Constant	17.353	13.532	1.645	0.200	

Comparisons between the groups preoperatively and one year postoperatively are summarized in Table 3. All factors except knee flexion angle in the good Δflexion group illustrated significant improvement from preoperatively to one year postoperatively.

**Table 3:** Comparisons between the groups preoperatively and one year postoperatively Abbreviations: ADL: activities of daily living; BMI: body mass index; FTA: femorotibial angle; IKES: isometric knee extension strength; KOOS: knee injury and osteoarthritis outcome score; QOL: quality of life a The paired t-test, b Wilcoxon signed-rank test.

Variables	Preoperation	One year postoperatively	p-value
Good flexion group (n = 38)			
FTA, °	180.42 ± 8.17	172.08 ± 3.14	0.001^b^
Rest pain, mm	20.63 ± 25.89	10.97 ± 17.88	0.032^b^
Walking pai, mm	51.97 ± 30.84	17.74 ± 21.02	0.001^b^
Knee flexion angle, °	127.24 ± 16.09	130.11 ± 11.97	0.053^a^
Knee extension angle, °	−3.18 ± 5.49	1.54 ± 6.07	0.001^a^
IKES, kgf	16.16 ± 10.56	18.63 ± 8.78	0.019^b^
KOOS, %			
Symptoms	64.38 ± 19.8	77.26 ± 12.6	0.001^a^
Pain	54.9 ± 22.81	78 ± 15.8	0.001^b^
ADL	62.89 ± 19.68	76.01 ± 14.17	0.001^a^
Rec/Sports	28.16 ± 24.89	43.82 ± 25.51	0.009^b^
QOL	34.54 ± 22.07	53.62 ± 18.36	0.001^b^
Poor flexion group (n = 41)			
FTA, °	182.59 ± 6.12	173.37 ± 2.86	0.001^b^
Rest pain, mm	16.17 ± 15.41	9.32 ± 19.96	0.002^b^
Walking pain, mm	48.63 ± 24.78	13.12 ± 21.16	0.001^b^
Knee flexion angle, °	141.32 ± 9.40	123.85 ± 11.58	0.001^b^
Knee extension angle, °	−0.64 ± 8.74	5.05 ± 7.40	0.001^b^
IKES, kgf	16.10 ± 8.77	20.12 ± 10.25	0.001^b^
KOOS, %			
Symptoms	62.2 ± 17.09	81.18 ± 16.83	0.001^b^
Pain	53.93 ± 16.59	85.16 ± 14.28	0.001^b^
ADL	63.49 ± 15.77	84.54 ± 12.78	0.001^b^
Rec/Sports	27.68 ± 24.06	49.27 ± 29.3	0.001^b^
QOL	33.23 ± 19.64	60.06 ± 24.76	0.001^b^

## Discussion

TKA has been increasingly performed as a therapeutic modality for patients with osteoarthritis recently. Among the many implant designs, the Attune CSRP TKA verified in this study demonstrated that the flexion position insertion of the femoral component was the factor that most affected the postoperative flexion angle improvement. By inserting the femoral component in a slightly flexed position, 1) the posterior femoral condyle osteotomy is reduced and the PCO is increased, 2) the bone volume in front of the distal femur is decreased, (Figure 3) the sliding surface between the posterior condyle and the insert is secured even in the deep flexed position. Previous reports that referred to sagittal alignment after TKA did not mention the femoral component; however, various reports have mentioned the PCO [6]. A larger PCO has been reported to be necessary to prevent the paradoxical roll-forward motion during flexion. The Low Contact StressTM (LCS: DePuy Synthes, Warsaw, IN) and Attune CSRP, which have different conformities between the femoral component and insert in flexion, are considered to have different ideal alignments based on the results of this study. The conformity ratio of TKA from extension to flexion gradually decreases [21]. However, this value was assumed to be balanced, including the difference between the medial and lateral sides of the implant gap in the extension and flexion positions. For all subjects in this study, we performed surgery to minimize gap imbalance with the gap balancing technique. Based on the results of this study, the preoperative ROM was worse in the poor Δflexion group than in the good Δflexion group as the patient background. The good Δflexion group included many patients with significant knee joint deformities and osteophytes. Therefore, invasive procedures such as osteophyte removal and soft tissue release were needed during surgery in many cases. As a result, this seems to have led to a decrease in the KOOS one year postoperatively. The second effect of flexion insertion of the femoral component, a decrease in anterior femoral offset, is associated with a decrease in the patellofemoral (PF) pressure from extension to flexion. Tanikawa et al. [22] also reported that PF pressure is not only determined by the thickness of the patella but also by the anterior-posterior positional relationship of the femoral component, and that the anterior flange should be placed in contact with the anterior cortex as much as possible. Because none of the subjects in this study underwent patellar replacement, the PF pressure in the flexed position was considered to be affected only by the thickness of the anterior distal femur. A previous study reported that PF pressure affects the femoral external rotation angle during surgery, and it has been reported that the greater the external rotation angle, the lower the PF pressure and the avoidance of anterior knee pain [23]. In this study, there was no significant difference in the intraoperative external rotation angle between the good and poor Δflexion groups, suggesting that the flexion angle was affected by the sagittal alignment of the femoral component. Scott et al. [24] reported that the insertion of a CR-type single-radius design femoral component in a flexed position reduced the incidence of anterior knee pain even in long-term results after TKA without patellar resurfacing.

Another advantage is the maintenance of the sliding surface between the femoral component and the insert in deep flexion. Regarding tibiofemoral sagittal conformity, it is known that Attune CSRP gradually decreases conformity from extension to flexion [21]. The insertion of the femoral component in the flexion position is considered to provide higher tibiofemoral joint conformity, even in deep flexion. Consequently, stress concentration on the insert is less likely to occur, even in the flexed position, and polyethylene wear is prevented, leading to aseptic loosening and revision [25]. It should be noted that depending on the posterior slope angle of the tibial component, insertion of the femoral component in a flexed position is a technique that can only be handled by CSRP, which does not have a post-cam mechanism like the PS type. The risk of post-delamination or breakage increases when the sum of the flexion angle of the femoral component and the posterior tibial slope angle exceeds the specified hyperextension allowable angle for models with a post-cam mechanism [26]. Laoruengthana et al. [27] reported that even after TKA, the extension ROM gradually widened until two years after surgery. For this reason, we should consider the difference in ideal alignment between the PS and cruciate-sacrificing (CS) types when selecting a model. Although the CSRP type has no particular restrictions on the sagittal alignment of the femoral component, excessive flexion insertion may cause postoperative flexion contracture [28]. In particular, it has been reported that males are significantly more likely to develop postoperative flexion contracture of 5° after TKA than females are [29].

Our findings may be useful for patients who require greater postoperative flexion ROM. In recent years, with increasing healthy life expectancy, the number of patients who require extensive activities, such as walking, cycling, golfing, and swimming, after TKA is increasing [30]. TKA is required not only to alleviate pain but also to maintain higher-order functions. The relationship between ROM after TKA and PROMs in this study is important for enhancing patients’ activity postoperatively.

Limitations

Although there was no difference in FTA between the good and poor Δflexion groups, factors related to intraoperative invasion, such as the level of deformity and the size of the osteophytes, have not been verified. The allowable range of femoral component flexion insertion has not yet been determined. The decrease in PF pressure due to femoral flexion insertion is theoretical only and has not been quantified. It is necessary to compare the thickness of the component with the actual osteotomy thickness from the anterior to the distal femur in the future. These are short-term results 1-year postoperatively; long-term follow-up data are not available. The sample size of 79 is not sufficient for a comparative study, and it is necessary to consider further increases in the number of subjects in the future.

The insertion of the femoral component in a flexed position is difficult to control using conventional techniques and requires computer-assisted surgery, such as navigation and robotics, to obtain a more accurate alignment. Since the grouping in this study was not the absolute value of the postoperative flexion angle but the difference from the preoperative period, there was a significant difference in ROM of the preoperative flexion of good and poor Δflexion groups. All patients underwent postoperative rehabilitation following a similar protocol; however, voluntary training after discharge was not uniform and could not be controlled.

## Conclusions

We found that the flexion insertion of the femoral component was an important factor in obtaining a larger flexion angle in the Attune CSRP. However, the hypothesis that the KOOS one year after surgery would be higher with better flexion ROM was overturned. It is necessary to identify factors other than ROM in postoperative satisfaction.
